# Utility of Transthoracic Echocardiography in Diagnostic Evaluation of Ischemic Stroke

**DOI:** 10.3389/fneur.2020.00103

**Published:** 2020-02-18

**Authors:** Jennifer Harris, Jason Yoon, Mohamed Salem, Magdy Selim, Sandeep Kumar, Vasileios Arsenios Lioutas

**Affiliations:** ^1^Department of Neurology, Beth Israel Deaconess Medical Center, Boston, MA, United States; ^2^Department of Neurosurgery, Beth Israel Deaconess Medical Center, Boston, MA, United States

**Keywords:** transthoracic echocardiography, ischemic stroke, diagnostics, stroke diagnosis, stroke etiology, resource utilization

## Abstract

**Objective:** Transthoracic echocardiography (TTE) is routinely performed as part of standard acute ischemic stroke (AIS) workup. However, the overall yield of TTE is unclear and many patients may undergo unnecessary investigations. This study aims to investigate the utility of TTE as part of AIS workup.

**Methods:** We collected data on consecutive patients with AIS who were admitted to our institution between 07/01/2016 and 09/30/2017. Patients were included based on neuroimaging-documented AIS, age >18 and neuroimaging studies. Primary endpoint was the proportion of cases in which TTE yielded relevant finding, defined as Atrial Septa Defect or Patent Foramen Ovale, left atrial enlargement, left ventricular thrombus or ejection fraction of <35%. Secondary endpoint was the proportion of patients who had a TTE-drive change in management.

**Results:** Among 548 AIS patients (median age 71 [59–81] years, 50% female), 482 (87%) underwent TTE. Clinically relevant findings were observed in 183 (38%) patients, leading to additional workup in 41 (8.5%). Further workup was associated with younger median age (58 [50–65] vs. 72 [62–81], *p* < 0.0001, and was less likely in suspected large vessel etiology (*p* = 0.02). Abnormal TTE lead to treatment change in 24 (5%) patients; 22/24 were started on anticoagulation. TTE results were less likely to influence treatment changes in older patients (71 [60–80] vs. 58 [49–69] years, *p* = 0.02) with known atrial fibrillation (*p* = 0.01).

**Conclusion:** Our findings suggest that despite widespread use, the overall yield of TTE in AIS is low. Stratifying patients according to their likelihood of benefitting from it will be important toward better resource utilization.

## Introduction

Identification of the etiology of acute ischemic stroke (AIS) is crucial for selecting optimal secondary preventative strategies. Since up to 30% of AIS can be attributed to a cardiac source (1), a cardiac workup including transthoracic echocardiography (TTE) is routinely performed to evaluate for clinically relevant findings including structural defects, left sided thrombi, or features suggestive of atrial fibrillation, such as enlarged left atrial appendages (2, 3). However, there are no clear guidelines regarding the utility of echocardiography in AIS, especially given the lack of data on how management changes are made based on TTE results. The European Stroke Organization guidelines recommend the use of echocardiography only in selected patients, such as patients with evidence of cardiac disease on history, examination, or electrocardiogram (ECG), suspected cardiac source of embolism (e.g., infarctions in multiple cerebral or systemic arterial territories), suspected aortic disease or paradoxical embolism and for patients with no other identifiable causes of stroke) (4). On the other hand, the American Stroke Association (ASA) up until now made no specific recommendation (5, 6). The recently released in the 2018 AHA/ASA guidelines take a more direct stance and explicitly advise against routine use of echocardiography in patients with AIS (7). These recommendations have led to significant uncertainties for practitioners and have implications for cost reimbursements; the guidelines do not specify which patients are likely to benefit from TTE which is was the rationale for this study. We sought to investigate the utility of TTE as part of AIS workup with a retrospective study investigating the frequency of abnormal echocardiography findings and TTE-driven change in management.

## Methods

This was a single-center retrospective observational study of prospectively collected data on consecutive patients with AIS who were admitted to a tertiary academic center in the USA, between 07/01/2016 and 09/30/2017. The study was approved by the local institutional review board. Informed consent was not applicable. Patients were selected based on the following inclusion and exclusion criteria: Patients with confirmed acute ischemic stroke, age >18 years old and with available neuroimaging studies. Patients were excluded if sudden onset focal neurological deficits resulted from an entity other than ischemic stroke, including intracranial hemorrhage, trauma/concussion, brain tumors, seizures, migraines, encephalitis, cerebral venous thrombosis, or metabolic derangements and if there was lack of baseline imaging data.

We collected and analyzed the following clinicodemographic variables: age, sex, race, cardiovascular comorbidities (hypertension, diabetes, hypercholesterolemia, history of coronary artery disease (CAD), atrial fibrillation (AF), congestive heart failure (CHF), chronic kidney disease (CKD), prior stroke or transient ischemic attack. We also collected information on prior use antiplatelets, anticoagulants, statins and antihypertensive medications as well as laboratory testing obtained routinely as part of AIS workup: Hemoglobin A1c, total cholesterol, triglycerides, low density lipoprotein (LDL) and high-density lipoprotein (HDL). We reviewed brain imaging obtained as routine AIS work up. All patients received brain MRI, except for those with contraindication, who received CT scan only. Also, all patients with suspected AIS received head and neck vessel imaging, either CT or MR Angiography.

All patients ≤60 years of age had a bubble study. In older patients, a bubble study was performed on a case by case basis according to the preferences of the individual Vascular Neurologist. Data abstraction was performed by TTE report review. All TTE were read by trained cardiologists, blinded to the patients' neurologic status and stroke subtype. Data abstracted from the report view included findings deemed to be of potential diagnostic relevance. These included (1) left atrial enlargement, (2) patent foramen ovale (PFO)/atrial septal defect (ASD), (3) depressed ejection fraction (EF) (defined as EF of <35%) (8), intracardiac thrombus and valve vegetation or other valvular abnormality.

Stroke subtypes were classified into five categories based on etiology, using the TOAST classification (9), modified to include the more recently evolved concept of the embolic stroke of undetermined source (ESUS) (10): (1) large-artery atherosclerosis (LAA), (2) small vessel occlusion (SVO), (3) cardioembolism (CE), (4) stroke of other determined etiology, (5) ESUS. LAA was defined as brain imaging findings of either significant (>50%) stenosis or occlusion of a major brain artery or branch cortical artery, presumably due to atherosclerosis, and a stroke in its downstream supplied territory. SVO was defined as a lesion diameter of <20 mm (11) and located in a subcortical area or brainstem on DWI MRI. The ESUS category was defined as non-lacunar infarct following specific imaging criteria: either cortical infarct or, for subcortical infarcts, a diameter of ≥1.5 cm on CT or ≥2.0 cm on DWI MRI (12). In addition the absence of large vessel atherosclerosis by CTA or MRA, lack of documented atrial fibrillation during hospital or outpatient long-term cardiac monitoring and CE made less likely by echocardiography were mandated (12). CE was defined as patients with arterial occlusions presumably due to an embolus arising in the heart. This was based on brain imaging findings which are similar to those described for large-artery atherosclerosis, but without significant (>50%) stenosis or occlusion of a major brain artery or branch cortical artery as well as brain imaging findings of stroke in multiple vascular territories, and cardiac source for an embolus based on history of AF, systemic embolism, or TTE findings suggestive of cardiac etiology. Neuroimaging was reviewed independently by two neurologists blinded to TTE findings.

The primary endpoint was the proportion of cases in which TTE yielded a diagnostically relevant finding as defined above. Our secondary endpoint was the number of patients who had a change of management due to an abnormal TTE finding, which was defined as one of two categories: (a) further work up (transesophageal echocardiography, MRV of the pelvis, lower extremity Doppler ultrasound) and (b) treatment change, either the form of medication change or intervention (initiation of anticoagulation, PFO closure, cardiac surgery).

## Statistical Analysis

Categorical and ordinal variables are presented as percentages and continuous variables as either mean ± standard deviation (SD) or median and interquartile range (IQR) depending on normality of distribution. Normality of distribution for continuous variables was tested with the Shapiro-Wilk test. We explored univariable associations between clinical, demographic and imaging variables and our outcomes of interest (clinically relevant TTE finding, TTE-driven additional workup and TTE-driven medication change or intervention). We used the chi-squared test for categorical variable comparisons. For continuous variables we used Student's *t*-test or the non-parametric Mann-Whitney U test depending on normality of distribution. Subsequently we constructed multivariable logistic regression models, including variables with statistical significance in the univariable models (defined as two-sided *p* < 0.05) and calculated odds ratios and 95% confidence intervals. Given that stroke phenotyping into certain subtypes might have been affected by TTE findings, we built two multivariable models, one excluding stroke subtypes and a second including stroke subtypes, if they had reached statistical significance in the univariable analysis. Analyses were performed in JMP Pro 12 (SAS, Cary, NC, USA).

### Baseline Cohort Characteristics

We identified 548 patients with AIS; 273 (49.8%) female, median age 71 (59–81) years. The baseline characteristics are summarized in Table 1. Our cohort comprised largely White (357, 66%) patients with the most common etiologies being CE (200; 36.5%) followed by LAA (127, 23.2%). Four hundred eighty-three (88%) patients received TTE as part of AIS workup. Differences between those who received TTE vs. those who did not are summarized in Table 1. Patients who received TTE were younger (70 [59–80] vs. 77 [64–86.5] years; *p* = 0.02), less likely to have AF (92 [19%] vs. 20 [31%]; *p* = 0.03) and receive anticoagulation therapy (73 [15%] vs. 17 [26%]; *p* = 0.03) and more likely to have received intravenous thrombolysis (93 [19%] vs. 5 [8%]; *p* = 0.02) and mechanical thrombectomy (44 [9%] vs. 1[1.5%]; *p* = 0.03).

**Table 1:** Summary of baseline cohort characteristics.

**Clinicodemographic characteristics**	**Entire cohort, *N* = 548**	**No TTE 65 (11.9%)**	**TTE 483 (88.1%)**	***p*-value**
Age, median (IQR)	71 (59–81)	77 (64–86.5)	70 (59–80)	0.02
Female sex, *n* (%)	273 (49.8)	37 (57)	237 (49)	0.23
**Race**				0.09
White	357 (66)	51 (78)	306 (63.5)	
African American	79 (14)	4 (6)	75 (15.5)	
Hispanic	22 (4)	4 (5)	19 (4)	
Asian	12 (2)	2 (3)	10 (2)	
Other/Unknown	78 (14)	6 (8)	72 (15)	
**Cardiovascular comorbidities**				
Hypertension, *n* (%)	401 (73.1)	49 (75)	352 (73)	0.76
Diabetes, *n* (%)	174 (31.7)	19 (29)	155 (32)	0.67
Hypercholesterolemia, *n* (%)	289 (52.7)	32 (49)	257 (53)	0.54
Current smoking, *n* (%)	90 (16.5)	11 (17.2)	79 (16.5)	0.88
CHF, *n* (%)	45 (8.2)	5 (8)	40 (8)	0.87
Afib, *n* (%)	112 (20.4)	20 (31)	92 (19)	0.03
Coronary artery disease, *n* (%)	97 (17.7)	10 (15.4)	87 (18)	0.73
Prior Stroke or TIA, *n* (%)	77 (14)	5 (8)	72 (15)	0.13
CKD, *n* (%)	57 (10.4)	10 (15)	47 (10)	0.16
**Laboratory values**				
Hemoglobin A1C, median (IQR)	5.7 (5.3–6.7)	5.8 (5.45–6.3)	5.7 (5.3–6.7)	0.32
Total Cholesterol, mean (±SD)	161 (133.5–197.5)	158 (138–184)	163 (133–198.75)	0.55
Triglycerides, mean (±SD)	115 (84–155.5)	116 (84.5–159)	115 (84–154	0.75
HDL, mean (±SD)	45 (37–57)	44.5 (34.75–61)	45 (37–56)	0.74
LDL, mean (±SD)	90 (63.5–117)	85 (68–101.5)	90.5 (63–119)	0.5
**Stroke subtypes**				0.53
Large artery atherosclerosis, *n* (%)	127 (23.2)	13 (20)	114 (24)	0.64
Cardioembolic, *n* (%)	200 (36.5)	20 (31)	180 (37)	0.39
Small vessel/lacunar	79 (14.4)	10 (15)	69 (14)	0.85
ESUS	91 (16.6)	13 (20)	78 (16)	0.48
Other defined causes/crytptogenic	51 (9.3)	9 (14)	42 (9)	0.18
**Medications**				
Anticoagulation on presentation, *n* (%)	90 (16.4)	17 (26)	73 (15)	0.03
Antiplatelets on presentation, *n* (%)	266 (48.5)	27 (42)	239 (50)	0.4
Statins on presentation, *n* (%)	283 (51.6)	33 (51)	250 (52)	0.89
Antihypertensives, *n* (%)	378 (68.9)	47 (72)	331 (69)	0.66
**Acute treatment**				
tPA given, *n* (%)	98 (17.8)	5 (8)	93 (19)	0.02
Mechanical thrombectomy, *n* (%)	45 (8.2)	1 (1.5)	44 (9)	0.03

### TTE With Findings of Potential Clinical Relevance

Echocardiographic findings of potential clinical significance were observed in 183 (38%) patients. The most frequent finding was left atrial enlargement, observed in 112 (23%) patients, followed by depressed EF (35 [7%] patients), PFO/ASD (35 [7%] patients), valve vegetations and other valvular abnormalities (10 [2%] patients) and intracardiac thrombus (5 [1%] patients). Characteristics of patients with and without clinically relevant findings are summarized in Table 2. In multivariable adjusted models, not including stroke subtypes, coronary artery disease (OR 1.95, 95% CI [1.21–3.15]; *p* = 0.006) and chronic kidney disease (OR 1.95, 95% CI [1.05–3.63]; *p* = 0.03) remained independently associated with higher odds of observing a clinically relevant echocardiographic finding. When including stroke subtypes in the multivariable model, presence of coronary artery disease remained associated with higher likelihood of clinically relevant finding (OR 1.9, 95% CI [1.17–3.11]; (0.01); conversely, LAA subtype was associated with lower odds (0.48 [0.27–0.81]; *p* = 0.007) (Table 2).

**Table 2:** Comparisons between patients according to relevant findings on TTE.

**Variables**	**No relevant findings 299 (62%)**	**Relevant findings 183 (38%)**	***p*-value**	**Multivariable logistic regression model 1^a^**		**Multivariable logistic regression model 2^b^**	
				**OR (95% CI)**	***p*-value**	**OR (95% CI)**	***p*-value**
Age, median (IQR)	71 (60–80)	70 (59–80)	0.91				
Female sex, *n* (%)	159 (53)	78 (42)	0.02	0.7 (0.48–1.02)	0.07	0.67 (.45–0.98)	0.04
**Race**			0.44				
White	185 (62)	121 (66)					
African American	52 (17)	23 (13)					
Hispanic	11 (4)	8 (4)					
Asian	8 (3)	2 (1)					
Other/Unknown	43 (14)	29 (16)					
**Cardiovascular comorbidities**							
Hypertension, *n* (%)	213 (71)	139 (76)	0.3				
Diabetes, *n* (%)	92 (31)	63 (34)	0.43				
Hypercholesterolemia, *n* (%)	156 (52)	101 (55)	0.56				
Current smoking, *n* (%)	45 (15)	34 (19)	0.37				
CHF, *n* (%)	22 (7)	18 (10)	0.35				
Afib, *n* (%)	52 (17)	40 (22)	0.28				
Coronary artery disease, *n* (%)	41 (14)	46 (25)	0.002	1.95 (1.21–3.15)	0.006	1.9 (1.17–3.11)	0.01
Prior Stroke or TIA, *n* (%)	50 (17)	22 (12)	0.19				
CKD, *n* (%)	22 (7)	25 (14)	0.02	1.95 (1.05–3.63)	0.03	1.79 (0.95–3.41)	0.07
**Laboratory values**							
Hemoglobin A1C, median (IQR)	5.7 (5.3–6.7)	5.7 (5.3–6.9)	0.67				
Total Cholesterol, mean (±SD)	167 (136–202.5)	158 (128–196)	0.051				
Triglycerides, mean (±SD)	115 (83–154)	113 (86–156)	0.91				
HDL, mean (±SD)	46.5 (37–58)	43 (35–54)	0.03				
LDL, mean (±SD)	92 (66–121)	88 (60–115)	0.09				
**Stroke subtypes**			<0.0001				
Large artery atherosclerosis, *n* (%)	88 (29)	26 (14)	0.0001			0.48 (0.27–0.81)	0.007
Cardioembolic, *n* (%)	90 (30)	90 (49)	<0.0001			1.52 (0.98–2.36)	0.06
Small vessel/lacunar	47 (16)	22 (12)	0.29				
ESUS	52 (17)	26 (14)	0.37				
Other defined causes	22 (7)	20 (11)	0.19				
**Medications**							
Anticoagulation on presentation, *n* (%)	37 (12)	36 (20)	0.03	1.69 (1.01–2.82)	0.04		0.2
Antiplatelets on presentation, *n* (%)	157 (53)	82 (45)	0.08				
Statins on presentation, *n* (%)	152 (51)	98 (53)	0.63				
Antihypertensives, *n* (%)	204 (68)	127 (69)	0.89				
**Acute treatment**							
tPA given, *n* (%)	62 (21)	31 (17)	0.3				
Mechanical thrombectomy, *n* (%)	27 (9)	17 (9)	1				

a Multivariable model including variables that reached statistical significance (p-value < 0.05) in univariable analyses excluding stroke subtype.

b *Variables included in model 1 plus stroke subtypes (if they reached statistical significance in univariable comparisons)*.

### TTE Leading to Additional Diagnostic Workup

Forty-one (8.5%) patients had additional workup as a result of a TTE finding. The most common additional diagnostic test was lower extremity venous Doppler ultrasound, in 25 (5%) patients, followed by transesophageal echocardiogram (TEE) in 17 (3.5%) patients, and MR Venogram of the pelvis in 16 (3.3%) patients. Characteristics of patients with and without additional TTE-related diagnostic workup are presented in Table 3. Patients who underwent additional testing were younger (58 [50–65] vs. 72 [61.5–81]; *p* < 0.0001), less likely to have hypertension (21 [51%] vs. 331 [75%]; *p* = 0.001) and AF (3 [7%] vs. 89 [20%]; *p* = 0.045) (Table 3). In multivariable adjusted models excluding stroke subtypes, only age retained its significant, inverse association with odds of receiving further workup (OR 0.94, 95% CI [0.92–0.97]; *p* < 0.0001). When including stroke subtypes, age retained the same association with unaltered effect size, while in addition, LAA stroke was independently associated with lower likelihood of receiving further workup (OR 0.17, 95% CI [0.03–0.59]; *p* = 0.003) (Table 3).

**Table 3:** Comparisons between patients according to the need of subsequent work up.

	**No additional workup 441 (91.5%)**	**Subsequent workup 41 (8.5%)**	***p*-value**	Multivariable logistic regression model^*^ 1^a^		Multivariable logistic regression model^*^ 2^b^	
				**OR (95% CI)**	***p*-value**	**OR (95% CI)**	***p*-value**
Age, median (IQR)	72 (61.5–81)	58 (50–65)	<0.0001	0.94 (0.92–0.97)	<0.0001	0.95 (0.92–0.97)	<0.0001
Female sex, *n* (%)	220 (50)	17 (41)	0.3				
**Race**			0.27				
White	284 (64)	22 (54)					
African American	65 (15)	10 (24)					
Hispanic	16 (4)	3 (7)					
Asian	10 (2)	0					
Other/Unknown	66 (15)	6 (15)					
**Cardiovascular comorbidities**							
Hypertension, *n* (%)	331 (75)	21 (51)	0.001	0.59 (0.29–1.22)	0.16	0.63 (0.3–1.32)	0.22
Diabetes, *n* (%)	142 (32)	13 (32)	0.96				
Hypercholesterolemia, *n* (%)	240 (54)	17 (41)	0.1				
Current smoking, *n* (%)	73 (17)	6 (15)	1				
CHF, *n* (%)	39 (9)	1 (2)	0.1				
Afib, *n* (%)	89 (20)	3 (7)	0.045	0.69 (0.16–2.14	0.28	0.6 (0.13–1.94)	0.42
Coronary artery disease, *n* (%)	84 (19)	3 (7)	0.09				
Prior Stroke or TIA, *n* (%)	68 (15)	4 (10)	0.49				
CKD, *n* (%)	44 (10)	3 (7)	0.58				
**Laboratory values**							
Hemoglobin A1C, median (IQR)	5.7 (5.3–6.7)	5.6 (5.4–6.9)	0.78				
Total Cholesterol, mean (±SD)	161 (132–198)	172 (140.5–208)	0.3				
Triglycerides, mean (±SD)	115 (84–154)	110 (83.5–159.5)	0.76				
HDL, mean (±SD)	45 (37–56)	46 (37–60)	0.66				
LDL, mean (±SD)	90 (63–116)	98.5 (70–125.75)	0.18				
**Stroke subtypes**			0.02				
Large artery atherosclerosis, *n* (%)	112 (25)	2 (5)	0.002			0.17 (0.03–0.59)	0.003
Cardioembolic, *n* (%)	164 (37)	16 (39)	0.87				
Small vessel/lacunar	63 (14)	6 (15)	1				
ESUS	66 (15)	12 (29)	0.025			1.31 (0.59–2.83)	0.49
Other defined causes	37 (8)	5 (12)	0.38				
**Medications**							
Anticoagulation on presentation, *n* (%)	69 (16)	4 (10)	0.3				
Antiplatelets on presentation, *n* (%)	227 (51)	12 (29)	0.008				
Statins on presentation, *n* (%)	234 (53.06)	16 (39)	0.09				
Antihypertensives, *n* (%)	310 (70)	21 (51)	0.02				
**Acute treatment**							
tPA given, *n* (%)	85 (19)	8 (20)	0.96				
Mechanical thrombectomy, *n* (%)	41 (9)	3 (7)	1				

* *Outcome of interest: Need for subsequent workup*.

a *Multivariable model including variables that reached statistical significance (p-value < 0.05) in univariable analyses excluding stroke subtype*.

b *Variables included in model 1 plus stroke subtypes (if they reached statistical significance in univariable comparisons)*.

### Echocardiographic Findings Leading to Treatment Change

Twenty-four (5%) patients had treatment change as a direct result of a TTE finding. Twenty-two (4.5%) commenced anticoagulant therapy, while two (0.4%) had a PFO closure and cardiac surgical procedure, respectively (Table 5). Characteristics of patients without vs. with treatment change are summarized in Table 4. Younger age (58 [49–69] vs. 71 [60–80]; *p* = 0.0003, presence of diabetes (13 [54%] vs. 142 [31%]; *p* = 0.02) and CE stroke (16 [67%] vs. 164 [36%]; *p* = 0.004) were all associated with higher likelihood of treatment change. Conversely, no patients with LAA and small vessel/lacunar strokes had treatment changes (Table 4).

**Table 4:** Comparisons between patients according to TTE role in treatment change.

	**No treatment change 458 (95%)**	**Treatment change 24 (5%)**	***p*-value**	Multivariable logistic regression model^*^ 1^a^		Multivariable logistic regression model^*^ 2^b^	
				**OR (95% CI)**	***p*-value**	**OR (95% CI)**	***p*-value**
Age, median (IQR)	71 (60–80)	58 (49–69)	0.0003	0.95 (0.92–0.98)	0.004	0.95 (0.92–0.98)	0.003
Female sex, *n* (%)	230 (50)	7 (29)	0.04	1.89 (0.78–5.1)	0.53	1.89 (0.74–5.27)	0.19
**Race**			0.71				
White	290 (63)	16 (67)					
African American	70 (15)	5 (21)					
Hispanic	19 (4)	0 (0)					
Asian	10 (2)	0 (0)					
Other/Unknown	69 (15)	3 (12)					
**Cardiovascular comorbidities**							
Hypertension, *n* (%)	336 (73)	16 (67)	0.48				
Diabetes, *n* (%)	142 (31)	13 (54)	0.02	2.7 (1.14–6.54)	0.9	3.33 (1.33–8.62)	0.01
Hypercholesterolemia, *n* (%)	245 (53)	12 (50)	0.75				
Current smoking, *n* (%)	76 (17)	3 (12)	0.78				
CHF, *n* (%)	38 (8)	2 (8)	0.99				
Afib, *n* (%)	91 (20)	1 (4)	0.05	0.29 (0.016–1.54)	0.54	0.12 (0.006–0.66)	0.01
CAD, *n* (%)	80 (17)	7 (29)	0.17				
Prior stroke or TIA	68 (15)	4 (17)	0.8				
CKD, *n* (%)	45 (10)	2 (8)	0.81				
**Laboratory values**							
Hemoglobin A1C, median (IQR)	5.7 (5.3–6.6)	6.2 (5.6–9.2)	0.007				
Total Cholesterol, mean (±SD)	113 (84–154)	144 (88.5–162)	0.25				
Triglycerides, mean (±SD)	115 (84–154)	110 (83.5–159.5)	0.76				
HDL, mean (±SD)	45 (37–57)	41 (37.5–51)	0.23				
LDL, mean (±SD)	91 (63–119)	79.5 (61–125.5)	0.81				
**Stroke subtypes**			0.002				
Large artery atherosclerosis, *n* (%)	114 (25)	0	0.002			Unstable term	0.003
Cardioembolic, *n* (%)	164 (36)	16 (67)	0.004			3.14 (1.19–8.97)	0.02
Small vessel/lacunar	69 (15)	0	0.04			Unstable term	0.01
ESUS	72 (16)	6 (25)	0.25				
Other defined causes	40 (9)	2 (8)	1				
**Medications**							
Anticoagulation on presentation, *n* (%)	71 (16)	2 (8)	0.33				
Antiplatelets on presentation, *n* (%)	227 (50)	12 (50)	0.97				
Statins on presentation, *n* (%)	236 (51)	14 (58)	0.51				
Antihypertensives, *n* (%)	314 (69)	17 (71)	0.81				
**Acute treatment**							
tPA given, *n* (%)	88 (19)	5 (21)	0.84				
Mechanical thrombectomy, *n* (%)	42 (9)	2 (8)	1				

* Outcome of interest: Treatment change.

a *Multivariable model including variables that reached statistical significance (p-value < 0.05) in univariable analyses excluding stroke subtype*.

b *Variables included in model 1 plus stroke subtypes (if they reached statistical significance in univariable comparisons)*.

**Table 5:** Types of treatment change.

**Type of treatment change**	**No. (%)**
Initiation of anticoagulation	22 (4.5)
Initiation of cardiac meds	0
PFO closure	2 (0.4)
Cardiac surgical procedure	2 (0.4)

## Discussion

In our study population 38% of patients had clinically relevant TTE findings, and approximately 9% necessitated additional workup, which ultimately resulted in treatment change in 5%. While there appears to be redundancy regarding TTE as part of AIS work up, there are at least some patients, who appear to benefit from TTE. Patients who were most likely to benefit from TTE were younger, had cardioembolic stroke subtype, or had a history of cardiac disease. Patients who were least likely to benefit from TTE were older patients with established AF, and patients with LAA and lacunar/ SVO stroke etiology.

Until recently, there were no clear guidelines regarding the utility of TTE in AIS (5, 6). In the most recent revision of the American Heart Association guidelines for the early management of patients presenting with AIS, did the ASA make firm recommendations against the routine use of TTE in AIS, which has sparked controversy as there is no elaboration on which patient subgroups might benefit from TTE. The new recommendation eliminates the perceived need for TTE in all AIS patients, that had elevated the routine TTE use into “standard of care,” which has very likely been a contributing factor to clinician overuse. On the other hand, many practicing clinicians have expressed concern that the updated recommendations adopt a simplistic stance that lacks granularity to appropriately stratify patients according to the likelihood of substantial benefit from TTE. In a challenging fiscal environment with health care organizations trying to reduce health care costs and prioritizing value and efficiency, performing a large number of redundant diagnostic tests will inevitably be targeted as wasteful. A more productive way of resource utilization would be to identify the patients that are much more likely to benefit, channel TTE usage toward them and educating physicians about targeted TTE use. Previous studies have shown that educating staff about the low yield of TTE in AIS resulted in a more cautious utilization and an overall decline in ordering echocardiograms (13).

TTE is performed with the intent to identify structural cardiac abnormalities of clinical relevance. Our study showed that clinically relevant findings are indeed relatively frequent, seen in 1/3 of patients. Our findings suggest that patients with LAA stroke subtype are the least likely to have a clinically relevant finding, suggesting that it might be reasonable to forgo TTE in this patient population. The importance of some of the TTE findings seems unequivocal; presence of valvular vegetation or intraventricular thrombus in the setting of multifocal acute infarcts would seal the diagnosis and management decision. Conversely, the pertinence of other findings is not entirely clear. This pertains in particular to left atrial enlargement which was the most common finding, seen in over 20% of our cohort. Traditionally, it has been viewed as indirect marker of occult atrial fibrillation (2, 3) although anticoagulation therapy is not advisable in the absence of confirmed atrial fibrillation. However, emerging evidence suggests that atrial enlargement, might be a biomarker of underlying thrombogenic atrial cardiopathy independent of atrial fibrillation with independent risk of recurrent cryptogenic or cardioembolic stroke (14, 15). The clinical relevance of this concept will be tested in the ongoing multicenter Phase III Atrial Cardiopathy and Antithrombotic Drugs In Prevention After Cryptogenic Stroke (ARCADIA) trial (NCT03192215), in which left atrial enlargement is being used among other inclusion criteria.

PFO is an additional finding that has sparked controversy in the past. Its high prevalence in the population (16), uncertainty regarding its implication as a causative factor and lack of efficacy with PFO closure (17) had limited the practicality of identifying a PFO. However, improved risk-stratification schemes (18) and findings from three recent randomized controlled trials (19–21) suggesting long-term benefit for PFO closure in select patients have of renewed interest in PFO detection. The most relevant question is whether TTE is practical as a useful PFO detection tool. It is known that TTE has high specificity but low sensitivity (22) and, indeed, in our cohort TTE identified a PFO only in 7% of the patients which is much lower than the prevalence reported in the literature (23, 24). On the other hand, TEE and transcranial Doppler, both of which have significantly higher sensitivity (22), present other limitations, including invasiveness, cost, need for specially trained personnel. Therefore, use of TTE as an initial screening tool in selected patients with embolic-appearing strokes and lack of other obvious stroke etiologies might be a reasonable strategy.

A more compelling estimate of the utility of routine TTE is whether it results in additional workup or therapeutic intervention. The proportions of patients were 8.5 and 5%, respectively which reveal that a considerable proportion of the studies performed might indeed be redundant. Although it might be difficult to identify patients likely to derive therapeutic or diagnostic benefit from TTE, it seems that at the very least it is feasible to identify patients whose management is least affected by TTE results. In our cohort none of the LAA and SVO stroke subtype patients had additional diagnostics or treatment change based on a TTE finding. This suggests that in patients whose strokes have been reliably phenotyped into these stroke subtypes, performing a TTE might be less impactful. Notably this simple observation applies to >1/3 of our patients who had a TTE, suggesting that with careful selection of target patients, the widespread TTE use could be appropriately curtailed.

Limitations of our study include a single center study design, as well as possibly practice bias, with younger patients prone to receiving both, more frequent further diagnostic testing and treatment change. For the etiologic classification we used the widely used TOAST classification. A different, more granular scheme, such as the *Causative Classification System for Ischemic Stroke (CCS)* might have yielded different distributions of stroke etiologies. Given that this might alter our estimation regarding the contribution of TTE in stroke diagnosis, it merits further elucidation in future studies (25). Our findings do not address the potential utility of TTE in managing post-stroke cardiorespiratory complications, which is driven by different, often critical clinical indications (26). There is also an inherent practical difficulty stemming from the fact that the intervention under study (TTE) is used, to some extent, to define some of the explanatory variables (stroke subtypes). At least two of the stroke categories (LAA and SVO) are defined based on brain and vascular imaging with minimal interference from TTE. We attempted to minimize it by including two different adjusted models, one including stroke subtype and one without; the adjusted model without stroke subtype is essentially agnostic to and unaffected by the TTE finding. In addition, our main outcome of interest (clinically relevant structural findings) is not affected by stroke subtype. However, this ultimately remains an important limitation, but one that is impossible to completely eliminate.

In conclusion, there might be redundancy in TTE utilization as part of AIS work up, but certain patients derive critical, treatment-defining benefit based on TTE findings. We must better define who these patients are, or at least start by identifying who are the least likely to benefit. Our study suggests that patients with LAA stroke subtype were the ones that consistently showed to have the least benefit from TTE. We consider our findings hypothesis-generating and do not advocate for limiting TTE use at the moment; external validation in different and larger cohorts including cost effectiveness analysis is needed.

## Data Availability Statement

Data that is not available with the article will be provided in an anonymized form by the corresponding author upon reasonable request from any qualified investigator. Indeed, if requested (e.g., for the purpose of a meta-analysis) data can be provided according to local IRB practices.

## Ethics Statement

The Institutional Review Board of Beth Israel Deaconess Medical Center approved the study, but waived the need for approval for this study protocol as it is retrospective.

## Author Contributions

JH: design and conceptualization of the study, analysis and interpretation of the data, drafting the original manuscript. JY and MSa: data collection, revising the manuscript for intellectual content. SK: revising the manuscript for intellectual content. MSe: revising the manuscript for intellectual. VL: design and conceptualization of the study, analysis and interpretation of the data, revising the manuscript for intellectual content.

### Conflict of Interest

The authors declare that the research was conducted in the absence of any commercial or financial relationships that could be construed as a potential conflict of interest.
